# Supplementary material for: Impact of Levetiracetam Treatment on 5-Aminolevulinic Acid Fluorescence Expression in IDH1 Wild-Type Glioblastoma
Source: Cancers (Basel). 2022 Apr 25;14(9):2134. doi: 10.3390/cancers14092134 (PMC9099986; doi:10.3390/cancers14092134)
Supplement: Supplementary file 1 [file cancers-14-02134-s001.zip › cancers-1675210-supplementary.pdf]

cancers

MDPI

Supplementary Materials

Impact of Levetiracetam Treatment on 5-Aminolevulinic Acid Fluorescence Expression in IDH1 Wild-Type Glioblastoma

Johannes Wach, Ági Güresir, Motaz Hamed, Hartmut Vatter, Ulrich Herrlinger and Erdem Güresir

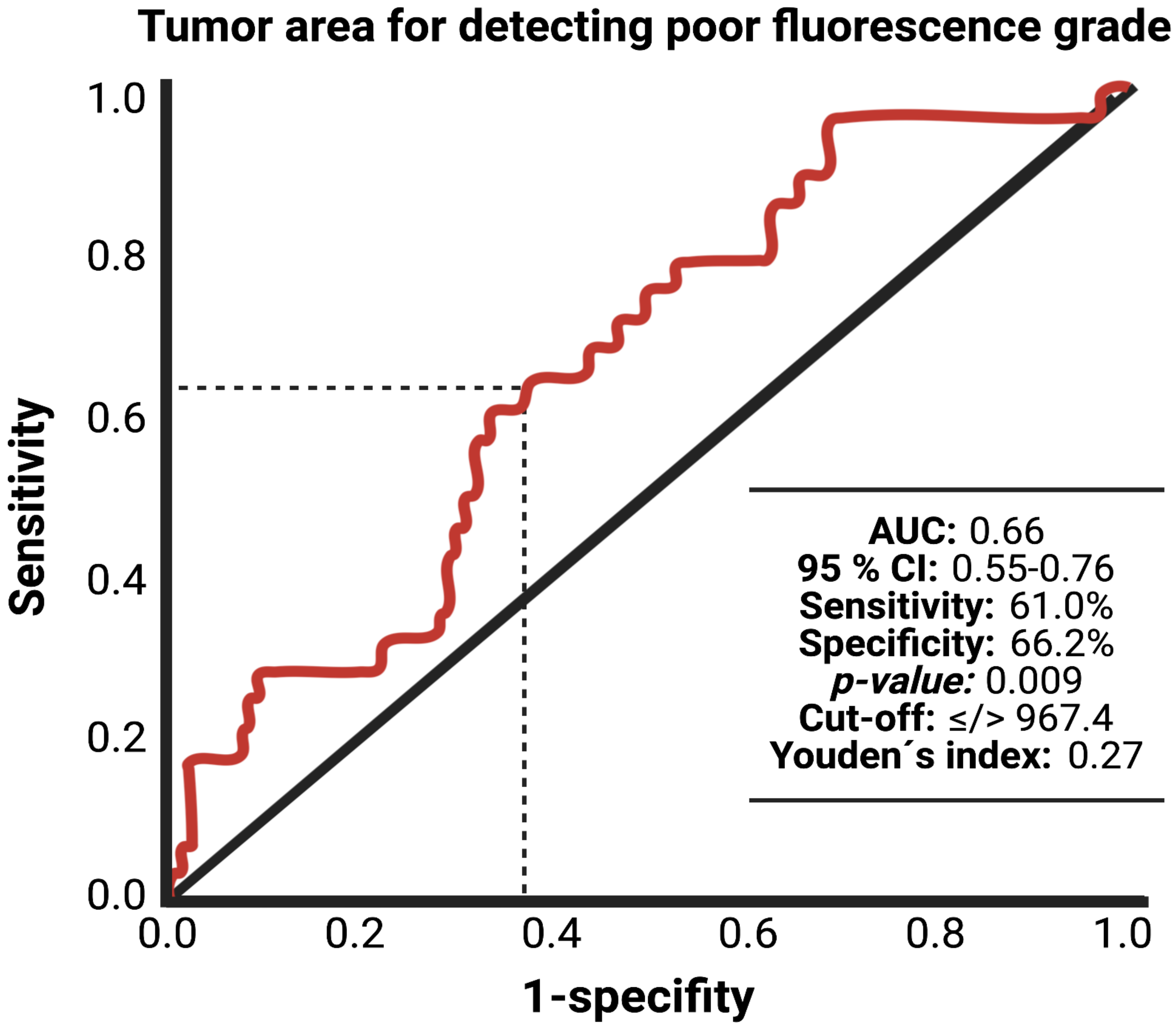

Figure S1. Receiver-operating characteristic curve illustrating tumor area in the prediction of poor fluorescence (fluorescence grades 0 + 1).
